# Maternal Prenatal Hair Cortisol Is Associated with Child Wheeze among Mothers and Infants with Tobacco Smoke Exposure and Who Face High Socioeconomic Adversity

**DOI:** 10.3390/ijerph18052764

**Published:** 2021-03-09

**Authors:** Ashley Scherman, Eliot R. Spindel, Byung Park, Robert Tepper, David W. Erikson, Cynthia Morris, Cindy T. McEvoy

**Affiliations:** 1 Department of Pediatrics, Oregon Health & Science University, Portland, OR 97239, USA; mcevoyc@ohsu.edu; 2 Oregon National Primate Research Center, Oregon Health & Science University, Beaverton, OR 97006, USA; spindele@ohsu.edu (E.R.S.); erikson@ohsu.edu (D.W.E.); 3 Biostatistics Shared Resource, the Knight Cancer Institute, Oregon Health & Science University, Portland, OR 97201, USA; parkb@ohsu.edu; 4 Herman B. Wells Center for Pediatric Research, Pediatric Pulmonology, Department of Pediatrics, Indiana University School of Medicine, Indianapolis, IN 46202, USA; rtepper@iu.edu; 5 Department of Medical Informatics and Clinical Epidemiology, Oregon Health & Science University, Portland, OR 97239, USA; morrisc@ohsu.edu

**Keywords:** pregnancy, psychological stress, smoking, disparities, asthma, in utero

## Abstract

The association of co-occurring prenatal stress and tobacco exposures on childhood wheezing and asthma are not well established. In this study, we compared maternal prenatal hair cortisol concentration (HCC) to the maternal report of infant wheezing (y/n) in the first year of life among mother–infant dyads exposed to tobacco smoke and socioeconomic adversity. Data were obtained from the Vitamin C to Decrease Effects of Smoking in Pregnancy on Infant Lung Function study. Maternal adversity was defined by the level of education, household income, and health insurance provider. Hair was collected at delivery, representing average circulating third-trimester cortisol levels. HCC was log transformed and dichotomized into high/low cortisol groups that were placed into a multivariate model predicting wheeze. Subjects (*n* = 132) were primarily White with ≤high school education and receiving government-provided health insurance. Forty-five percent of infants wheezed. Average HCC was 3.39 pg/mg hair. Women with HCC > 3.55 pg/mg were more than twice as likely to report having a child who wheezed (odds ratio 2.56, 95% confidence interval 1.22–5.40; *p* = 0.01), adjusting for insurance provider and maternal asthma. Among this sample of dyads with prenatal smoke exposure, elevated maternal HCC was associated with child wheeze that was not diminished after consideration of covariates.

## 1. Introduction

Childhood wheezing and asthma disproportionately affect children who face adversity. Asthma prevalence among Black or African American children is 15.7% compared to non-Hispanic White children (7.1%) and 11.8% among children of families living at <100% of the federal poverty line compared to 7.4% of children of higher-income families [1]. Although prenatal maternal tobacco smoking is a well-established risk factor for childhood wheezing and asthma [2], evidence increasingly suggests that maternal prenatal psychological stress and correlates of stress (i.e., maternal depression and anxiety) contribute to childhood wheezing and asthma [3]. Women who report stressful life circumstances such as low income and limited educational attainment are also at higher risk of smoking cigarettes during pregnancy [4,5]. The relationship of these co-occurring prenatal exposures on childhood wheezing and asthma remain poorly elucidated.

Prenatal tobacco smoke exposure increases the risk of childhood wheezing and asthma [6,7]. A meta-analysis of 14 studies across 12 years found that maternal prenatal smoking carried a 40% increased risk of early childhood (≤2 years of age) wheezing and asthma (odds ratio [OR] 1.41, 95% confidence interval (CI) 1.20–1.67) [8]. Smoking is thought to increase the risk of wheezing and asthma directly by impacting lung development and function and indirectly by increasing the risk of premature birth and low birthweight, which are important determinants of lung function [9,10,11]. Fortunately, the prevalence of prenatal smoking over the past four decades has decreased, from 20% to 35% in the 1980s to 7.1% in 2016 [12,13]; however, reductions in prenatal smoking are not equally distributed across the US population.

Women who face social and economic adversity are at higher risk of prenatal smoking. Among 279,063 pregnant women, approximately 12.5% of those who reported smoking had less than or equal to a high school education compared to 0.8% of women with a bachelor’s degree or higher [12]. Although more non-Hispanic White women reported smoking (10.5%) than non-Hispanic Black women (6%) in this sample, when compared to the total population of US women who gave birth in 2016, non-Hispanic Black women were overrepresented. In 2016, 51% of births were among non-Hispanic White women compared to 15% among non-Hispanic Black women, suggesting that the proportion of non-Hispanic Black pregnant women who smoke is twice that of pregnant non-Hispanic White women. Additionally, women who report life stressors such as emotional, financial, and traumatic stress, and women who report depression, a correlate of stress, are more likely to smoke during pregnancy [14,15]. In a large sample of 178,937 pregnant women receiving Medicaid, life stressors were positively associated with the probability of prenatal smoking. Reporting just 1–2 life stressors increased the probability of smoking by 6% relative to reporting no life stressors [16]. In general, women with low income and low educational attainment are at increased risk for reporting perinatal stressful life events, psychological stress and correlates of stress, and depression and anxiety [17,18].

Maternal prenatal psychological stress has been linked to childhood wheezing and asthma. Van de Loo et al. [3] examined 10 observational studies of prenatal maternal stress and respiratory morbidity in childhood. The prevalence of respiratory symptoms was higher in children of mothers who were exposed to or experienced some form of psychological stress during pregnancy than in children of mothers who did not (OR 1.56, 95% CI 1.36–1.80). A later meta-analysis of 12 studies [19] also found an increased risk of childhood asthma in relation to maternal psychological stress indexed by acute and chronic life stressors and depression and anxiety (OR 1.15, 95% CI 1.04–1.27). Although highly suggestive, these reviews did not report on studies of biological measures of the stress response. Products of the maternal stress response such as adrenergic hormones and glucocorticoids (i.e., cortisol) have been shown to impact fetal lung growth and maturation [20], program the immune system [21], and/or alter gene expression; however, the results of human studies of the stress hormones are mixed. Wright and colleagues [22] measured maternal stress, defined as salivary cortisol levels over three days in mid-late gestation. Elevated evening cortisol was associated with increased odds of repetitive wheezing in the offspring through two years of life (OR 2.2, 95% CI 1.09–4.09, *n* = 261). Conversely, de Vries et al. [23] reported no association of a single first-trimester serum cortisol level with childhood wheeze among 2227 Dutch women (OR 1.00, 95% CI 0.99–1.00). The influence of maternal stress on childhood respiratory health is likely part of a complex interplay of prenatal exposures. Emerging evidence supports the modifying effect of maternal stress on environmental exposures and childhood wheezing. Lee et al. [24] and Rosa et al. [25] found that air pollution in an urban setting increased the risk of asthma only among children whose mothers experienced high prenatal stress suggesting a role for stress and environmental exposures on asthma; however, investigators typically control for maternal smoking as a confounding variable [3,19], thus the potential dual effects of maternal stress and tobacco smoke exposure on childhood wheezing and asthma remain unclear.

Given the potential adverse effects of co-occurring prenatal stress and tobacco smoke exposures on childhood wheezing and asthma disparities, our primary objective was to examine the relationship between maternal chronic stress, indexed by prenatal hair cortisol concentration (HCC), and maternally reported wheezing in children through 12 months of age among a sample of 132 US pregnant women exposed to prenatal cigarette smoke and high socioeconomic adversity. We hypothesized that HCC would remain significant despite maternal smoking.

## 2. Materials and Methods

### 2.1. Study Design

We analyzed data from the double-blind, randomized controlled intervention trial, Vitamin C to Decrease Effects of Smoking in Pregnancy on Infant Lung Function (VCSIP, NCT01723696). A brief description of VCSIP is provided below. A detailed description of the study design and primary outcomes are published elsewhere [26,27]. Between December 2012 and June 2015, 251 English-speaking pregnant women at least 15 years of age, with a singleton gestation, and who reported smoking cigarettes were recruited from clinics delivering at Oregon Health and Science University (OHSU) in Portland, OR, Peace Health Southwest Medical Center in Vancouver, WA, and from Indiana University and Wishard Hospital in Indianapolis, IN. Eligible participants were allocated to 500 mg/day vitamin C or placebo between 13^0/7^ and 22^6/7^ weeks of gestation. Secondary outcomes included a maternal report of child wheezing through 12 months of age. Participation in VCSIP was voluntary and written informed consent was obtained. The study was approved by the institutional review boards at the participating sites. Notably, women were provided pregnancy-specific smoking cessation pamphlets and counseled for smoking cessation at every interaction with VCSIP research personnel using the 5As: (1) ask women about tobacco use; (2) advise women of the importance of quitting; (3) assess willingness to stop; (4) assist women who are ready to quit; (5) arrange for follow up [28]. The study was designed with an intention to treat; a date was recorded for women able to quit smoking and they continued through study protocol completion.

#### 2.1.1. Maternal Cortisol

During the VCSIP study, hair samples were collected at delivery, initially for evaluation of nicotine exposure, and some hair was subsequently allocated for cortisol measurement. Hair was cut close to the root from the posterior aspect of the head and tied at the cut end to identify the most recent portion of hair growth. Circulating cortisol is incorporated into hair at approximately 1 cm per month, providing a measure of systemic cortisol levels over time [29,30]. We extracted cortisol from the proximal 3 cm in length segments of hair, considered representative of the prior three months of circulating cortisol levels (i.e., the last trimester) [29]. Hair cortisol levels were measured by the Endocrine Technologies Core at the Oregon National Primate Research Center as detailed in Appendix A. Briefly, hair quantity was limited (0.22 to 2.44 mg), thus we measured cortisol using a highly sensitive liquid chromatography-tandem triple quadrupole mass spectrometry (LC–MS/MS) assay. Approximately 5 hair strands were selected, cut to 3 cm in length from the root end, and weighed to the closest 0.01 milligram (mg). Samples were extracted into 2 mL methanol by overnight incubation in an ultrasonic bath at 55 °C then measured by LC–MS/MS on a Shimadzu Nexera-LCMS 8050 (Shimadzu). Data were processed and analyzed using LabSolutions Software, V5.72 (Shimadzu). The assay quantifiable range was 1.044 picograms (pg)/sample to 4275 pg/sample. This lower limit of quantitation in hair was 0.3 pg/mg. Intra-assay and inter-assay coefficients of variation were less than 6% (*n* = 3 assays).

#### 2.1.2. Maternal Smoking and Childhood Wheeze

Maternal smoking status was indexed as positive at baseline if a woman reported smoking at least one cigarette per day (CPD) in the week prior to enrollment and as positive during postpartum if a woman reported smoking at least one CPD on any respiratory questionnaire through their child’s first year of life.

The maternal report of wheeze was ascertained via standardized respiratory questionnaires (RQ) [31] administered quarterly by phone or in-person through 12 months of age as part of VCSIP. A positive report of wheeze was defined as any positive response to the question, “Since (birth/the last time we talked) has your child had wheezing or whistling in his/her chest?” at any time.

#### 2.1.3. Socioeconomic, Demographic, Correlates of Stress, and Health Factors

The VCSIP study collected maternal, labor, and delivery outcome data through interviews and electronic medical records. At enrollment, baseline characteristics were collected from maternal report unless otherwise indicated, and included maternal race; maternal education (<high school, high school diploma or General Educational Development (GED), some college, Bachelor’s degree); maternal age in years; type of insurance (government-provided, none or self-paid, or private insurance); household income during pregnancy (<$25,000, $25,000–$60,000, >$60,000); maternal relationship status (single, divorced, married, or significant other). In addition, maternal history of asthma, maternal depression or anxiety diagnoses, and maternal non-insulin-dependent diabetes diagnosis were all (y/n) ascertained from medical records). Maternal body mass index (BMI) is categorized following the Centers for Disease Control guidelines [32]. Maternal and infant characteristics ascertained at delivery from medical records included infant sex (male vs. female); gestational week (term (≥to 37th) vs. not term (<37th)).

### 2.2. Analysis

Baseline characteristics were summarized descriptively. Pearson chi-square and Wilcoxon rank sum were used to examine the relationships between wheeze and maternal and infant characteristics for categorical and continuous variables, respectively. HCC was evaluated for normality and log transformed for skewness then dichotomized into high and low groups using empirical receiver operating characteristics (ROC). Dichotomized hair cortisol values were evaluated by ROC characteristics because predictability was higher and more clinically relevant using this parameterization instead of the continuous parameterization. Based on ROC analysis, the log(HCC) value of 3.55 achieves the largest value of the Youden Index (sensitivity + specificity − 1). In other words, the log(HCC) value of 3.55 achieves the largest sensitivity plus specificity [33]. Dichotomized HCC was fit in a logistic regression model to predict the categorical wheeze outcome (Yes/No). These models were run both unadjusted and adjusted for potential confounding variables identified in univariate models with a significance *p*-value < 0.20. Additionally, an exploratory analysis tested associations between HCC quartiles and childhood wheeze using logistic regression models with the first quartile as the reference group. Analyses were performed using SAS 9.4 (SAS Institute Inc., Cary, NC, USA).

## 3. Results

A total of 251 women participated in the VCSIP study and hair samples for cortisol analysis at delivery and maternally reported wheeze were available for 180 participants. We included subjects with observations above the quantifiable lower limit of 1.04 pg/sample for a final sample size of 132. There was no appreciable difference in wheeze between dyads with a detectable maternal cortisol vs. those without (*p* = 0.76). Mothers were primarily White/Caucasian (74.2% White/Caucasian) and had low socioeconomic status (86.4% received government-provided health insurance or had no insurance and 26.5% reported a household income of <$25,000 per year). A total of 36% of mothers had a history of asthma and 45.5% of children experienced wheeze in the first year of life. Children who experienced wheezing were more likely to have mothers with a history of asthma (*p* = 0.06) and with government-provided insurance (*p* = 0.03). Over 35% of participants reported depression or anxiety although this was not associated with child wheezing (*p* = 0.13). All participants smoked prenatally and just two mothers reported not smoking or no CPD through 12 months after delivery; the amount of prenatal and postnatal smoking was not associated with wheeze (Table 1).

Mean log-transformed HCC (logHCC) was 3.39 pg/mg (standard deviation (SD) = 1.4; range = 0.9–8.9). Table 2 shows unadjusted and adjusted logistic regression model outputs for the relationship between maternal logHCC (continuous and dichotomized ≤3.55 pg/mg) and childhood wheeze. Cortisol was positively associated with childhood wheeze. Women with logHCC greater than 3.55 pg/mg were 2.83 times more likely to have a child who experienced wheeze (95% CI, 1.31–6.14; *p* = 0.008) adjusting for insurance, asthma, self-reported depression/anxiety, CPD in pregnancy and postpartum, and diabetes. The addition of adjustment variables did not appreciably change effect estimates. An association by quartile of logHCC was not consistently detected, but the quartile with the highest logHCC also had the greatest frequency of wheeze and the greatest measure of association.

## 4. Discussion

Among mother–infant dyads exposed to tobacco smoke and who face socioeconomic adversity, elevated maternal HCC in the last trimester of pregnancy was associated with child wheezing through 12 months of age (*p*-value = 0.008. Our findings are congruent with other studies showing an association between maternal prenatal psychological stress and childhood wheeze and asthma [3,19,22,23]. By design, we used measures of the biological stress response and our sample population was homogenous for perinatal tobacco exposure, which is a known contributor to childhood wheeze and asthma [7]. Despite the strong presence of competing predictors, the relationship between maternal hair cortisol and childhood wheeze supports a role for the maternal prenatal stress response in the development of childhood wheezing and asthma.

The VCSIP study did not capture maternal perceived stress, instead, we leveraged available behavioral and sociodemographic data to characterize chronic maternal life stressors. There was no association between CPD and infant wheezing; however, 45% of mothers in our sample reported infant wheezing compared to 27.5% from the most recent large study of maternal report of wheezing in the first year of life among 14,059 French families [34]. An often-cited but older report across a larger age range of 826 US families found that 33.5% of children reported wheezing in the first three years of life [35]. Both studies found maternal smoking increased the risk of childhood wheezing (OR 1.57 and 2.2, respectively), and thus our results likely reflect a higher overall burden of wheezing. Among measures of economic adversity, only maternal insurance status was associated with wheeze in our population. Most women had ≤a high school education, which is commonly linked to smoking risk [5]. Women primarily reported household yearly income above the 2013 poverty line (between $25,000 and $60,000); however, 25% reported household incomes below $25,000 which was higher than the national average of 14.5% for 2013 [36]. Reporting of private insurance (13% of the sample) was associated with wheeze and may reflect unmeasured social and economic support that affected mother–infant health (i.e., type of personal or partner employment). We did not find a significant relationship between Black/African American and child wheezing. The lack of a direct relationship between maternal race and childhood wheeze counters data from nationally representative samples, where Black/African American children in the lowest socioeconomic brackets are disproportionately burdened by asthma (adjusted OR 1.99, 95% CI 1.09, 3.64 (*n* = 14,244)) [37]. Most likely, we failed to detect a relationship due to our relatively small sample of dyads who identified as Black/African American (*n* = 22). A larger sample may elicit underlying differences in the relationship between stress and early life wheezing among mothers identifying as White versus those identifying as Black/African American [5].

Maternal history of asthma stood apart as a risk factor for infant wheeze (*p* = 0.06), which is expected based on previous work. Familial asthma risk may act on different mechanistic pathways than stress and smoking. In total, 64% of mothers in our sample reported depression or anxiety and this was not associated with wheeze. Identifying the prevalence of prenatal depression and anxiety nationally is challenging, although the evidence suggests that approximately 25% of women report psychological distress including depression or anxiety during pregnancy [17]. A relationship between depression and anxiety and wheeze may have been washed out due to the high prevalence of depression and anxiety in this sample or bias in self-reporting. Depression and anxiety were self-reported in our cohort and not ascertained using validated instruments which could have biased the frequency of depression/anxiety in our study. Anticipating the high prevalence of self-reported psychological distress, our team chose a biological measure of the stress response.

The mean logHCC among our study population was 3.39 pg/mg SD ± 1.4, which is comparable to reported HCC among pregnant populations utilizing similar methods. Approximate differences in mean logHCC between our results and others ranged from −2 to 1.28 pg/mg [38,39,40]. Limitations in our hair cortisol analysis exist. We measured cortisol in hair because our primary interest was the biological response to chronic stress. HCC is an average of circulating cortisol levels over time with greater concentration suggesting higher stress. However, cortisol secretion also follows a circadian rhythm and HCC does not capture these daily patterns of cortisol secretion, which may be important in studying the health effects of stress [41]. Future studies may benefit from evaluating both long-term and 24 h levels of cortisol. Hair samples were not available for all VCSIP participants and 27% of our hair samples returned cortisol concentrations that were below the limit of quantification (LOQ). No apparent pattern in hair processing emerged that could explain this phenomenon. Results below the LOQ were equally distributed across all batches of hair samples. Evidence exists for both positive and negative associations between circulating cortisol and chronic psychological stress [42]. Dyads excluded for HCC below the LOQ showed no difference in the maternal report of childhood wheeze. We did not consider whether hair maintenance such as dyes, other chemical treatments, or frequency of shampooing would decrease cortisol content, although reports on whether hair maintenance affects cortisol content in hair are mixed [30]. Finally, we had limited hair available for analysis. A maximum of 2.5 mg of hair per participant was available, and for some samples, less than 0.5 mg of hair was available for assay as allocation of hair samples for cortisol analysis was not a primary objective of the VCSIP trial [27]. Obtaining large quantities of hair can present challenges regardless of protocol agendas (e.g., women with thin hair may provide smaller samples) and refining methods for the analysis of cortisol from small quantities of hair would be advantageous.

## 5. Conclusions

In a cohort homogenous for maternal tobacco smoking, a known risk factor for childhood wheezing and asthma, rates of maternal reported wheezing were high and the relationship between maternal stress and offspring was not washed out, suggesting that exposure to high prenatal stress further influenced childhood respiratory outcomes; however, our results should be interpreted with caution as this is a secondary data analysis where data were not initially collected to meet the objectives and mediation analysis of prenatal stress on smoking exposure and infant wheeze was not completed. Further studies, where the primary objective is to compare biomarkers and women’s experience of chronic stress with childhood wheeze, are critical for the identification of dyads at the highest risk for wheezing and asthma and would be useful for the design of effective interventions.

## Figures and Tables

**Table 1:** Maternal and infant characteristics and wheeze.

		N (%) or Median (IQR)			
		Overall	No Wheeze	Wheeze	*p*-Value ^a^
Maternal		*n* = 132	*n* = 72	*n* = 60	
Age (years)		28 (23.5, 31.5)	28.5 (24.0, 32.0)	26 (22.0, 31.0)	0.21
Race	American Indian and Alaska Native	2 (1.5)	1 (1.4)	1 (1.7)	0.99
	Black/African American	22 (16.7)	12 (16.7)	10 (16.7)	
	More than one Race	10 (7.6)	6 (8.3)	4 (6.7)	
	White	98 (74.2)	53 (73.6)	45 (75.0)	
Insurance status	Government provided	112 (84.9)	56 (77.8)	56 (93.3)	0.03
	None or self-paid	2 (1.5)	1 (1.4)	1 (1.7)	
	Private insurance	18 (13.6)	15 (20.8)	3 (5.0)	
Household income	<$25,000	35 (26.5)	23 (31.9)	12 (20.0)	0.66
	$25,000–$60,000	57 (43.2)	27 (37.5)	30 (50.0)	
	>$60,000	7 (5.3)	5 (6.9)	2 (3.3)	
	Unknown and other *	33 (25.0)	17 (28.3)	16 (26.7)	
Marital status	Divorced	7 (5.3)	4 (5.6)	3 (5.0)	0.29
	Married	24 (18.2)	17 (23.6)	7 (11.7)	
	Significant other	56 (42.4)	30 (41.7)	26 (43.3)	
	Single	45 (34.1)	21 (29.2)	24 (40.0)	
Education	Bachelor’s degree	5 (3.8)	3 (4.2)	2 (3.3)	0.74
	High school or GED	44 (33.3)	21 (29.2)	23 (38.3)	
	Less than high school	29 (22.0)	17 (23.6)	12 (20.0)	
	Some college	54 (40.9)	31 (43.1)	23 (38.3)	
Asthma	No	84 (63.6)	51 (70.8)	33 (55.0)	0.06
	Yes	48 (36.4)	21 (29.2)	27 (45.0)	
Depression/anxiety	No	84 (63.6)	50 (69.4)	34 (56.7)	0.13
	Yes	48 (36.4)	22 (30.6)	26 (43.3)	
CPD baseline		7.0 (4.0, 10.0)	8.0 (4.0, 10.0)	5.0 (4.0, 10.0)	0.14
CPD postpartum ^b^		10 (5.0–10.0)	10.0 (5.0–10.0)	7.0 (5.0–10.0)	0.15
Diabetes	No	130 (98.5)	70 (97.2)	60 (100)	0.19
	Yes	2 (1.5)	2 (2.8)	0 (0)	
BMI pre-pregnancy		26.7 (22.6, 33.2)	25.9 (22.2, 32.4)	26.8 (23.3, 33.7)	0.39
Treatment group (vitamin C/placebo)	A	64 (48.5)	32 (44.4)	32 (53.3)	0.31
	B	68 (51.5)	40 (55.6)	28 (46.7)	
Child					
Term birth	No term	14 (10.6)	8 (11.1)	6 (10.0)	0.84
	Term	118 (89.4)	64 (88.9)	54 (90.0)	
Sex	Female	64 (48.5)	38 (52.8)	26 (43.3)	0.28
	Male	68 (51.5)	34 (47.2)	34 (56.7)	

IQR = interquartile range; CPD = cigarettes per day; BMI = body mass index (kg/m^2^); GA = gestational age; GED = general education diploma. ^a^ alpha level = 0.10. ^b^ *n* = 104. * unknown (N/A Not Collected, unknown).

**Table 2:** Maternal hair cortisol and childhood wheeze.

		No Wheeze N (%)	Wheeze N (%)	*p*-Value ^a^	Unadjusted OR (95% CI)		*p*-Value ^a,c^	Adjusted OR (95% CI) ^b^		*p*-Value ^a,b,c^
Log (HCC) > 3.55 pg/mg	No	52 (64.2)	29 (35.8)		ref			ref		
	Yes	20 (39.2)	31 (60.78)	0.005	2.78	(1.35–5.73)	0.006	2.83	(1.31–6.14)	0.008
HCC quartiles										
	1	18 (54.6)	15 (45.5)		ref			ref		
	2	25 (75.8)	8 (24.2)	0.071	0.38	(0.13–1.10)	0.074 ^c^	0.24	(0.08–0.78)	0.018 ^c^
	3	16 (48.5)	17 (51.52)	0.622	1.28	(0.49–3.35)	0.623	1.34	(0.48–3.74)	0.573
	4	13 (39.4)	20 (60.6)	0.218	1.85	(0.69–4.91)	0.219	1.49	(0.53–4.17)	0.453

CI = confidence interval; OR = odds ratio; HCC = hair cortisol concentration. ^a^ alpha level 0.05. ^b^ adjusted for insurance, asthma, self-reported depression/anxiety, CPD in pregnancy and postpartum, and diabetes. ^c^ *p*-values for exploratory logistic regression models for each respective quartile compared to the first quartile.

## Data Availability

Investigators in this study are aware of and will abide by the principles for sharing research resources described in the “Principles and Guidelines for Recipients of NIH Research Grants and Contracts on Obtaining and Disseminating Biomedical Research Progress.” Data may be shared upon request of Dr. Cynthia McEvoy and Dr. Cynthia Morris, consistent with privacy, security, and informed consent restrictions.

## Appendix A

We measured cortisol using a liquid chromatography-tandem triple quadrupole mass spectrometry (LC–MS/MS) assay. As described in the main document calibrators, quality controls (QCs), and hair samples were subjected to overnight extraction and transferred to microtiter plates. Microtiter plates were loaded onto a SIL-30ACMP autosampler (Shimadzu, Kyoto, Japan) and 25 µL of each sample, calibrator, or QC was injected onto a Raptor 2.7 µm Biphenyl 50 × 2.1 mm column (Restek, Bellefonte, PA, USA) at 40 °C using reversed-phase chromatography. Mobile phase A was 0.15 mm ammonium fluoride; mobile phase B was 100% methanol. The LC time gradient was created using two Nexera LC-30AD pumps (Shimadzu) as follows: 0.00–2.00 min 50%–72% B; 2.01–2.10 min 72%–100% B; 2.11–2.75 min 100% B; 2.76–3.00 min 100%–50% B; 3.01–4.60 min hold at 50% for re-equilibration prior to the next injection. The entire gradient was run at a flow rate of 0.4 mL/min. Heated electrospray ionization in the positive mode with scheduled multiple reaction monitoring (MRM) on a Shimadzu LCMS-8050 was used for the detection of cortisol. The interface temperature was 300 °C, desolvation line temperature was 150 °C, and the heat block temperature was 500 °C. Gas was supplied by a Peak Genius 1051 nitrogen and air generator (Peak Scientific, Inchinnan, UK). Nitrogen gas was used for nebulizing (3 L/min) and drying (10 L/min) gases; air (10 L/min) was used for heating gas. Argon (Airgas, Radnor, PA, USA) was used for collision-induced dissociation at 270 kPa. The tandem mass spectrometry (MS/MS) conditions for cortisol and cortisol-d4 were optimized using the automated MRM optimization procedure in LabSolutions software (Shimadzu). The MRM transitions for cortisol were: 363.10 > 121.20 (quantifying ion), 363.10 > 327.20 (reference ion), retention time 1.886 min. The MRM transitions for cortisol d-4 were: 367.15 > 121.20 (quantifying ion), 367.15 > 331.30 (reference ion), retention time 1.874 min. Data were then processed and analyzed using LabSolutions Software, V5.72 (Shimadzu) with final results reported in the main document.
